# Efficient WSI classification with sequence reduction and transformers pretrained on text

**DOI:** 10.1038/s41598-025-88139-5

**Published:** 2025-02-15

**Authors:** Juan I. Pisula, Katarzyna Bozek

**Affiliations:** 1 Institute for Biomedical Informatics, Faculty of Medicine and University Hospital Cologne, University of Cologne, Cologne, Germany; 2 Center for Molecular Medicine Cologne (CMMC), Faculty of Medicine and University Hospital Cologne, University of Cologne, Cologne, Germany; 3 Cologne Excellence Cluster on Cellular Stress Responses in Aging-Associated Diseases (CECAD), University of Cologne, Cologne, Germany

**Keywords:** Image processing, Computational science

## Abstract

From computer vision to protein fold prediction, Language Models (LMs) have proven successful in transferring their representation of sequential data to a broad spectrum of tasks beyond the domain of natural language processing. Whole Slide Image (WSI) analysis in digital pathology naturally fits to transformer-based architectures. In a pre-processing step analogous to text tokenization, large microscopy images are tessellated into smaller image patches. However, due to the massive size of WSIs comprising thousands of such patches, the problem of WSI classification has not been addressed via deep transformer architectures, let alone via available text-pre-trained deep transformer language models. We introduce SeqShort, a multi-head attention-based sequence shortening layer that summarizes a large WSI into a fixed- and short-sized sequence of feature vectors by removing redundant visual information. Our sequence shortening mechanism not only reduces the computational costs of self-attention on large inputs, it also allows to include standard positional encodings to the previously unordered bag of patches that compose a WSI. We use SeqShort to effectively classify WSIs in different digital pathology tasks using a deep, text pre-trained transformer model while fine-tuning less than 0.1% of its parameters, demonstrating that their knowledge about natural language transfers well to this domain.

Tranformers^1^ have brought several breakthroughs to the disciplines of natural language processing (NLP) and computer vision (CV). Their capacity to link information across sequences of vector embeddings, representing either visual features or vectorized words, allowed to capture the structure and meaning necessary for machine translation^2–5^, question-answering^6–9^, image classification^10–12^ and segmentation^11,13^, and even multi-modal tasks such as text-to-image generation^14,15^.

Concurrently in the field of digital pathology, the popularization of Multiple Instance Learning (MIL)^16,17^ approaches for Whole Slide Image (WSI) analysis allowed for the fast adoption of transformer models in this domain. By considering each WSI as a set of feature vectors of smaller tissue patches, this type of data is a natural input to transformer architectures. However, although transformer-based, these methods are typically modified and adapted to the idiosyncrasies of MIL and histopathology. Given gigapixel image size, out-of-the-box Vision Transformers (ViTs)^10^ are excessively memory-demanding. Diverse shapes of WSIs and removal of patches consisting of background, artifacts, such as pen marker lines, require tailored implementation of local or windowed attention^18,19^. Novel positional encoding methods have been proposed to replace fixed and learnable positional embeddings commonly found in NLP transformers or ViTs^20–25^. To overcome the challenges of WSI processing, we base our work on the two observations below.

- *The redundancy of information present in full-sequence self-attention operations* can be exploited to reduce the computational cost of large inputs in deep transformer models. Wang *et al.*^26^ base their Linformer model on the observation that an attention matrix can be approximated with a matrix of lower rank. The works of Liu *et al.*^11^ and Dai *et al.*^27^ propose to construct hierarchical representations instead of maintaining full-length, token-level resolution. The observations made by Clark *et al.*^28^ about the importance of the [SEP] token and neighboring tokens have inspired several methods of local and sparse attention^29–32^. Comprising thousands of image patches, a WSI representation in a MIL approach is a prohibitively long sequence of vector embeddings. *We hypothesise that such findings in the transformer literature are valid to histopathology data as well*, and techniques for attention matrix reduction are necessary to allow for processing of massive in size WSIs with the use of transfomers.
- *Text pre-trained transformers have been proven successful in non-language related tasks.* Recent works have shown that language models pre-trained on large unstructured text corpora not only perform strongly in various downstream NLP tasks, but in several tasks outside of this domain, ranging from solving math problems^33^, to lossless image and audio compression^34^. We refer to^35^ for an extensive enumeration of such works. In the context of CV, Ilarco *et al.*^36^ showed that text representations of frozen language models are predictive of visual representations of their corresponding object. More recently, Lu *et al.*^37^ demonstrated that pre-trained language models show high performance in image classification, numerical computation, and protein fold prediction when less than 0.1% of their parameters are fine-tuned. *Language-based pre-training can therefore be leveraged to perform different, out-of-domain tasks,* which however has never been demonstrated in WSI classification.

In this work we use deep transformer architectures to classify WSI data. To allow for processing of thousands of image patches from a single slide, we propose *SeqShort*, a multi-head attention (MHA) input layer that reduces long input sequence to a fixed-size short sequence that can be processed by any transformer model. Furthermore, we show that classification performance is increased when the transformer classifier is pre-trained with a language modeling task compared to training it from scratch, and that only fine-tuning less than 0.1% of its weights is necessary. This way, we construct a deep, yet computationally inexpensive model that requires a reduced set of trainable parameters, and performs well in digital pathology tasks.

## Results

We compress the visual information of WSIs with our sequence reduction technique and use transformer models trained from scratch or pre-trained on text data to solve several WSI classification tasks. We train multiple transformer architectures and find that text pre-training improves classification performance in deep transformer models. In our approach the input is in a form of an ordered sequence, instead of an unordered collection of image patches as commonly done in other MIL algorithms. We further show that positional information that we add to the ordered sequences is taken into account by the transformer classifier and improves its prediction accuracy.

We then examine how our SeqShort layer works to better understand how visual information in the WSIs is aggregated. We find that only a small subset of image patches per WSI is relevant to produce their compressed sequence representations, corroborating our hypothesis about information redundancy in WSIs. Although these representations act as potentially lossy summaries of the WSIs, an extension of the attention rollout algorithm^38^ can trace the output of the transformer classifiers back to each individual image patch, providing an interpretability mechanism for the classification outcome.

### WSI classification

**Table 1:** Performance of different MIL algorithms in the different slide-level classification tasks using EfficientNet features^43^.

Method	x10 magnification		x20 magnification	
	25% train set	100% train set	25% train set	100% train set
Lymph Node Metastases classification				
ABMIL^39^	0.501	0.664	*0.516*	0.616
CLAM^40^	0.511	0.692	*0.516*	0.673
DS-MIL^41^	0.468	0.695	0.441	0.640
TransMIL^20^	*0.529*	0.629	0.470	0.723
Wagner et al.^42^	0.465	**0.778**	0.501	*0.778*
Ours	**0.627**	*0.772*	**0.642**	**0.865**
Invasive Breast Carcinoma subtype classification				
ABMIL^39^	0.542 ± 0.107	0.571 ± 0.088	0.551 ± 0.103	0.554 ± 0.107
CLAM^40^	0.811 ± 0.055	0.850 ± 0.039	0.697 ± 0.056	0.791 ± 0.082
DS-MIL^41^	0.779 ± 0.075	0.892 ± 0.045	0.711 ± 0.084	0.819 ± 0.082
TransMIL^20^	*0.864 ± 0.063*	*0.896 ± 0.048*	**0.782 ± 0.094**	*0.856 ± 0.064*
Wagner et al.^42^	0.687 ± 0.202	0.854 ± 0.069	0.739 ± 0.099	0.824 ± 0.077
Ours	**0.874 ± 0.052**	**0.901 ± 0.049**	*0.765 ± 0.099*	**0.863 ± 0.047**
Renal Cell Carcinoma subtype classification				
ABMIL^39^	0.724 ± 0.077	0.795 ± 0.040	0.697 ± 0.077	0.758 ± 0.044
CLAM^40^	**0.965 ± 0.013**	0.969 ± 0.025	*0.961 ± 0.013*	0.974 ± 0.010
DS-MIL^41^	0.941 ± 0.047	0.971 ± 0.001	0.926 ± 0.025	0.963 ± 0.001
TransMIL^20^	*0.962 ± 0.015*	**0.980 ± 0.001**	**0.971 ± 0.010**	*0.980 ± 0.001*
Wagner et al.^42^	0.960 ± 0.020	*0.979 ± 0.009*	0.971 ± 0.011	**0.984 ± 0.007**
Ours	0.942 ± 0.019	0.974 ± 0.011	0.952 ± 0.017	0.977 ± 0.013

Best and the second best classification results are in bold and italics, respectively.

We measure the performance of our method on three different classification tasks: Lymph Node Metastases (LNM) classification (Normal vs Metastases); Invasive Breast Carcinoma (IBC) subtype classification (Invasive Ductal Carcinoma vs Invasive Lobular Carcinoma); and Renal Cell Carcinoma (RCC) subtype classification (Papillary Cell Carcinoma vs Chromophobe Cell Carcinoma vs Clear Cell Carcinoma). For the LNM classification task we use the dataset provided by the CAMELYON16 grand challenge (https://camelyon16.grand-challenge.org/), keeping 10% of the training samples as a validation set, and evaluating on the grand challenge test set. For the cancer subtyping tasks, we use WSIs collected from The Cancer Genome Atlas (TCGA) (https://www.cancer.gov/tcga), and follow the same stratified 10-fold cross-validation as^40,44^.

We use 256$\times$256 image patches cropped from the WSIs both at $\times 10$ and $\times 20$ magnification. As a data scarcity ablation, we train the models using the complete datasets or just 25% of the samples. Area under ROC curve (AUROC) is used as classification performance metric. We compare our method against several state-of-art weakly supervised architectures. All networks are compared using a single magnification at a time, and are agnostic of how their input features vectors were produced. We use an EfficientNetV2-L^43^ pre-trained on ImageNet^45^ as patch-level feature extraction network in this work. As our best performing model we use a frozen RoBERTa-base^8^ model as MIL classifier, and only fine-tune its normalization layers. Results of this experiment are shown in Table 1, and additional results of LNM and IBC classification using a CTransPath^46^ feature extractor are shown in the Supplementary Table 1.

Although 99.9% of the parameters in our model were trained solely on text data, it surpasses WSI-specific methods in most LNM and IBC experiments, while demonstrating competitive AUROC in RCC classification. The experiments utilizing the CTransPath feature extractor generally enhance the performance of all models, with a slight performance decline for our model only in the LNM task when using 25% of the data.

### Pre-training on text improves WSI classification

**Table 2:** Performance of different Language Models in IBC subtype classification, at $\times 20$ magnification.

Language Model	AUROC
Baseline	0.784 ± 0.082
XLNet-base^47^	0.819 ± 0.090
GPT2-small^48^	0.827 ± 0.079
BERT-base^6^	0.849 ± 0.058
ALBERT-base^7^	0.747 ± 0.118
Llama3-8B^49^	0.810 ± 0.070
RoBERTa-base^8^	**0.863 ± 0.047**

We explore the use of popular NLP transformer architectures that can be trained in a single GPU for sequence classification in the WSI classification task. Such architectures have not been applied before in weakly-supervised histopathology tasks given the computational cost of handling thousands of instances in a single WSI. The sequence shortening method that we introduce in this study allows us to overcome the computational cost problem.

Inspired by the success of pre-trained language models in different tasks outside the NLP, we propose the use of a frozen, language-modeling pre-trained transformer as MIL classifier. This is motivated by the hypothesis that the multi-head self-attention (MHSA) layers of a transformer language model learn to capture the interdependencies among the elements of sequences, independent of the original data modality or domain. We follow^37^ and only fine-tune the normalization layers of the model, reducing the amount of trainable parameters in our transformer encoder from 85M to 36K (only 0.04% of the total amount).

An important question is if text pre-training does play a role in classification performance. We compare the performance of a baseline transformer encoder trained from scratch with different frozen text-pre-trained transformers. Given our GPU memory constrains, the SeqShort layer was required in order to train these models. All the tested models have 12 layers of 12 attention heads and 768 hidden units, resulting in comparable transformer size across all models, except for Llama3-8B^49^. Llama3-8B is a larger, 8 billion parameter model comprising 32 layers of 32 attention heads and 4096 hidden units. The model was fine-tuned with 8-bit model weights to fit it in our hardware. The baseline model, BERT-base, and RoBERTa-base have identical architecture and only differ in text-pre-training dataset and language modeling task.

This experiment was done at $\times 20$ magnification, using the IBC dataset. Except for ALBERT-base^7^, every model outperforms the baseline (Table 2) indicating that pre-training on large corpus of text data does influence model performance in other domains including digital pathology.

### The role of positional information

**Table 3:** Effect of including positional information on classification performance of IBC subtyping.

Magnification	Pos. embedding		AUROC
	WSI	Seq.	
$\times 20$	No	No	0.825 ± 0.052
	No	Yes	0.863 ± 0.047
	Yes	No	0.865 ± 0.044
	**Yes**	**Yes**	**0.866** ± **0.064**
$\times 10$	No	No	0.884 ± 0.062
	No	Yes	0.901 ± 0.049
	Yes	No	0.916 ± 0.046
	**Yes**	**Yes**	**0.917** ± **0.035**

In our approach, we consider a WSI to be an unordered bag of image patches, and SeqShort provides positional information for free by reducing it to an ordered, fixed-length sequence of feature vectors. This enables the adding of the fixed-size set of learnable positional embeddings which is common practice in transformer architectures of CV and NLP tasks to the output of SeqShort.

Different positional encodings based on patch location have been proposed to address the problem of varying WSI shapes and sizes^20–25^, and their inclusion is compatible with our method. In this section, we repeat the IBC subtyping experiment to investigate the effect of positional information on classification performance. In this experiment, we enhance our classifier with the patch location positional encoding used in^23^ previous to the SeqShort input, in addition to the standard BERT positional embedding used before the transformer classifier.

The results of the experiment are shown in Table 3. In the unordered bag of patches formulation, the ordered output sequence of SeqShort carries positional information that can be exploited by adding positional encoding, increasing AUROC by 0.017 at $\times 10$ magnification and by 0.038 at $\times 20$ magnification. Positional encoding of the patches based on their 2D spatial location also improves performance, and the best results are achieved when both types of positional encoding are employed.

**Fig. 1:**
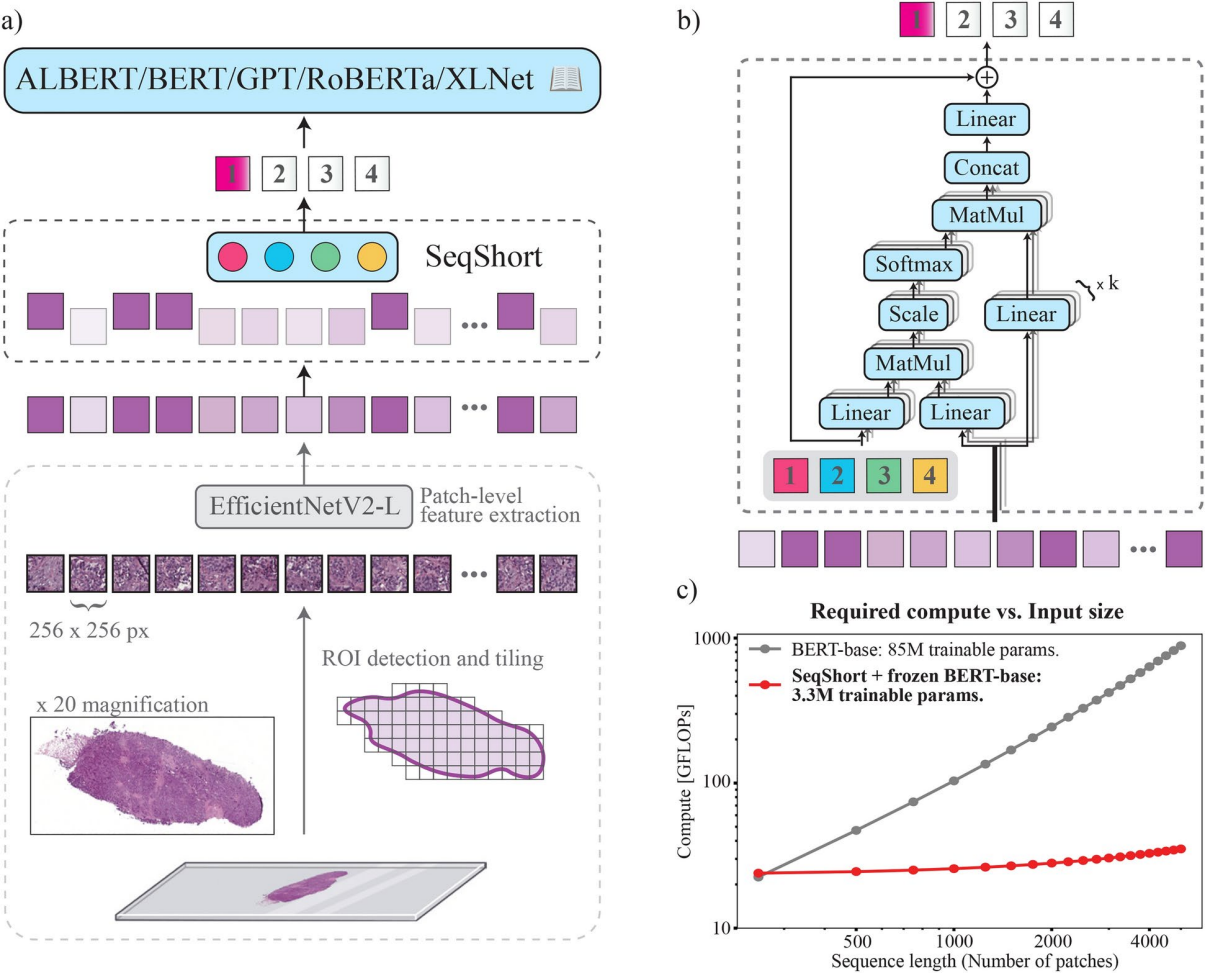
Proposed method. (**a**) From bottom upwards: after a typical MIL pre-processing step (tiling, feature extraction), our SeqShort layer using a pre-defined number of query vectors (colored circles) summarises the long list of patches into a small, ordered sequence of feature vectors (colored squares) which are then classified by a deep transformer model that was pre-trained on an extensive text corpus. Different patches are in varying proportion part of the resulting feature vectors which is symbolically represented by their color intensity. (**b**) Detailed view of the SeqShort layer, where a set of learnable vectors (colored squares) query the relevant information in the WSI patches via a multi-head attention operation. (**c**) The computational cost of a forward pass of a deep transformer classifier is considerably reduced when our SeqShort layer is used (measured with the fvcore library by FAIR^1^).

### Insights into sequence summarization

**Fig. 2:**
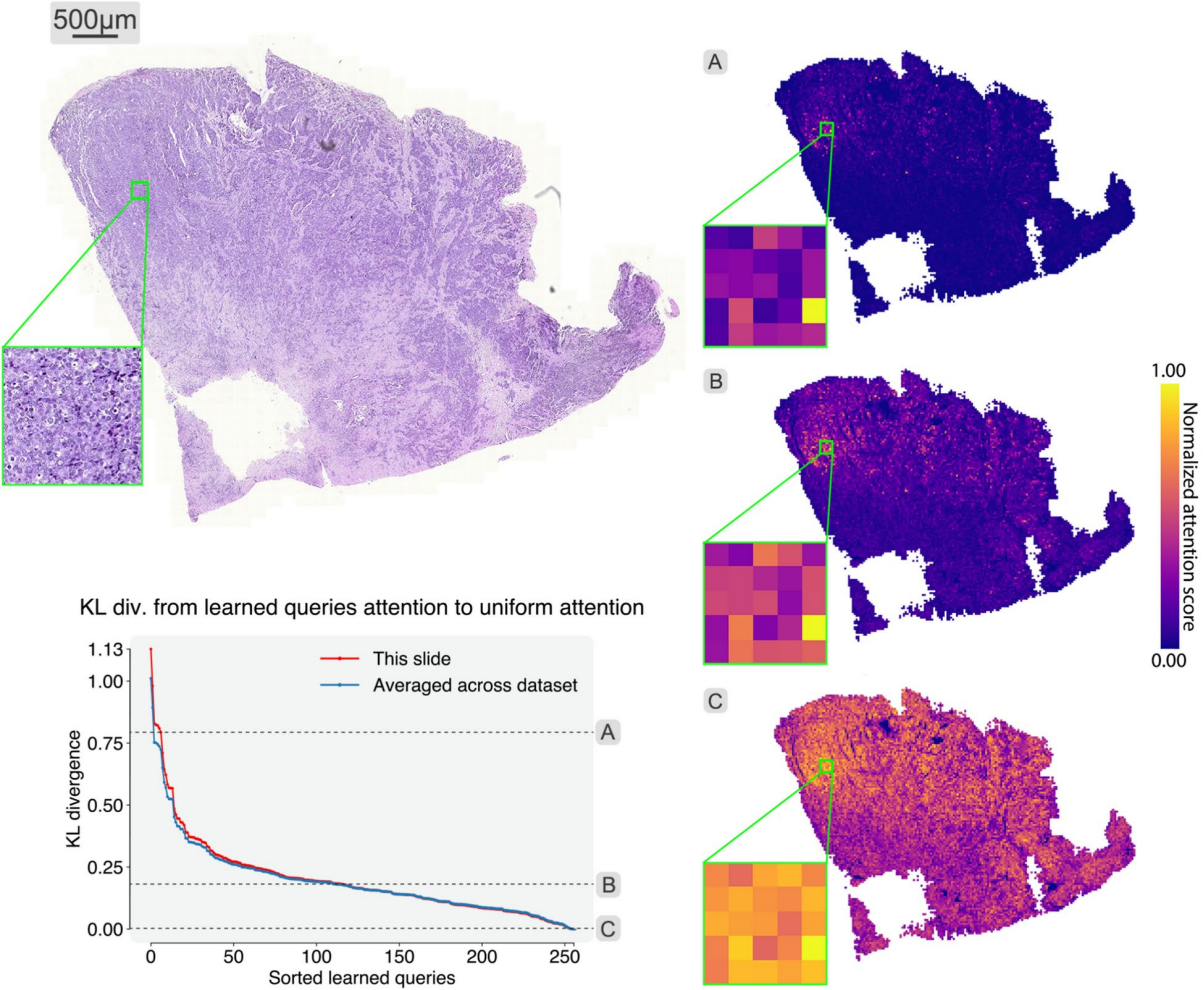
WSI summarization. A WSI, and attention heatmaps (**A**–**C**) produced by three different query vectors in SeqShort are shown. Although different queries show attention distributed over a broader or narrower set of patches, the most important instances agree among the heatmaps. The bottom left plot shows the Kullback-Leibler divergence from the attention distributions of the learned queries to uniform attention, and the values that correspond to the heatmaps are indicated with dashed lines. Values are sorted for ease of visualization, higher values correspond to uneven distribution of attention among patches.

We probe the SeqShort layer to examine how a WSI is summarized. We calculate the Kullback-Leibler (KL) divergence between the attention distributions produced by the different learned query vectors in SeqShort and a uniform attention distribution. Values close to zero indicate that such queries pay overall the same amount of attention to all the input patches, whereas higher KL divergence values suggest that such queries pay more attention to a reduced subset of image patches. We do this measurement with every sample of one of the IBC test sets at $\times$20 magnification, and average the results.

The KL divergence values are shown in Fig. 2, as well as an example WSI and the attention heatmaps produced by three different learned query vectors. The individual heatmaps demonstrate that indeed some patches receive more attention than the others. However across the three heatmaps, even though the attention distributions are spread over various-sized image areas, the same patches receive high-attention.

We confirm this visual insight by calculating the Spearman’s rank correlation coefficients between pairs of different learned query vectors’ ranking of patches (within a single WSI). For the WSI in Fig. 2 and the three examined query vectors, the correlation coefficients are above 0.96, and when considering the complete set of 256 query vectors, the mean rank correlation coefficient value is 0.99 (with a minimum value of 0.76). Among all the WSIs in the test set, 99.7% of the total pairs of rankings show a correlation coefficient > 0.7.

### Explanation of classification outcome

**Fig. 3:**
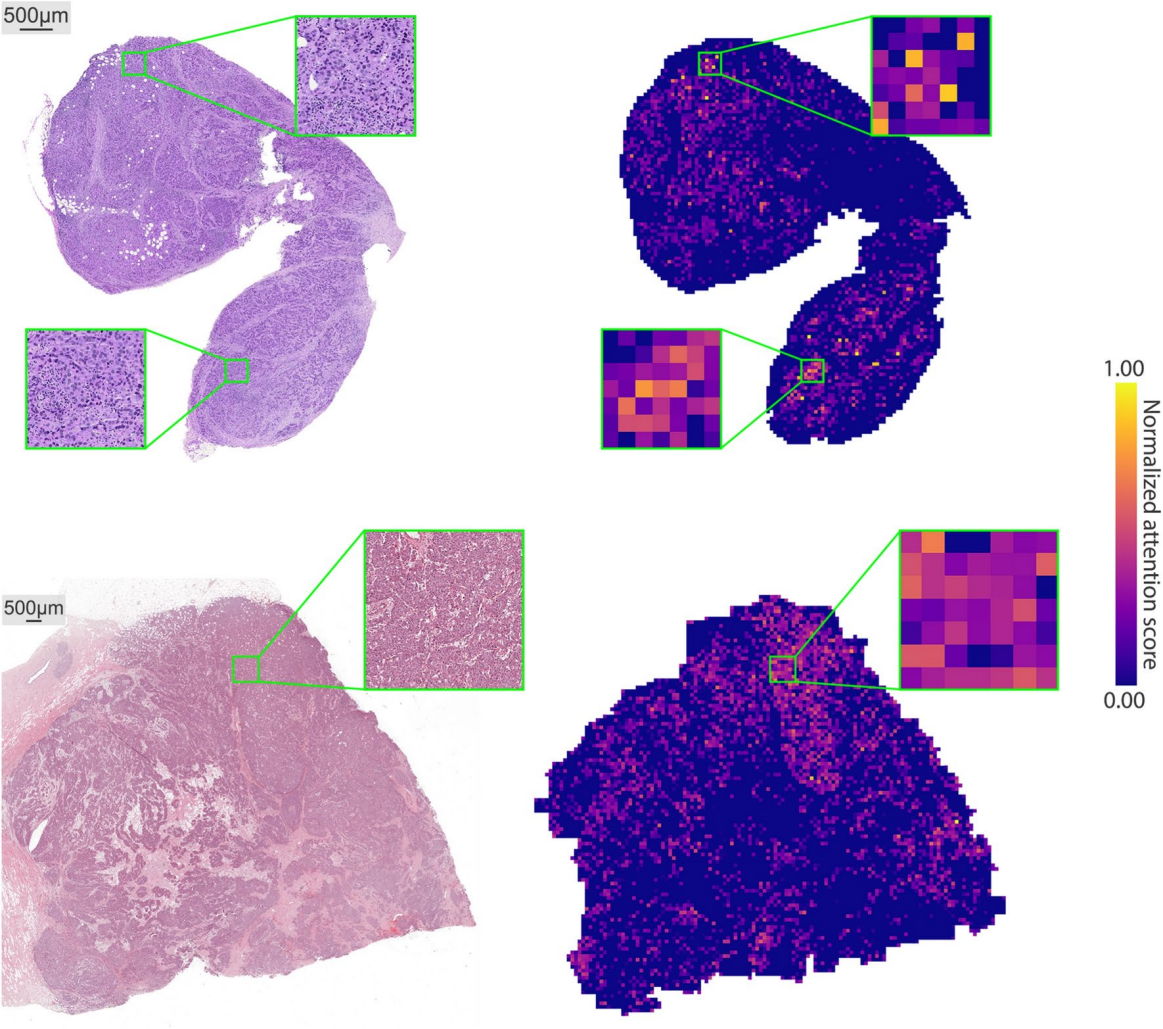
Attention rollout heatmaps. Left: original WSIs. Right: their corresponding attention rollout heatmaps. Although the SeqShort layer reduces the number of feature vectors that the downstream transformer has to process, it is still possible to backtrack the contribution of each individual image patch to the classification decision using this most common transformer explainability method.

Attention heatmaps from the previous experiment illustrate the functioning of the SeqShort layer of our model: they provide insights into how the individual patches of a WSI are weighed to synthesize the intermediate output of our method.

Given how the model aggregates the patch representations throughout its forward pass, we apply attention rollout^38^ to generate heatmaps that provide insights into the overall attention the model assigns to each patch in its decision process. We modify the base case of the recursive definition of attention rollout to take into consideration that SeqShort is the first layer of the complete model. Our modified attention rollout is then defined as:

1 $$\begin{aligned} \varvec{\tilde{A}_{i}} = {\left\{ \begin{array}{ll} \varvec{A_{i}} \cdot \varvec{\tilde{A}_{i-1}} & \text {if}\, i>0\\ \begin{bmatrix} \varvec{0} \\ \varvec{A_{i}} \end{bmatrix}&\text {if }i=0, \end{array}\right. } \end{aligned}$$

where $\varvec{A_{i}}$ is the attention matrix of layer *i*, and $\varvec{0}$ is the zero vector in row space, to take into account that the [CLS] token was not present in the MHA operation of SeqShort. Example heatmaps are shown in Fig. 3. Hence, while allowing to process large WSIs, the SeqShort mechanism does not limit the interpretability of the predictive model.

## Discussion

In this work we use a text pre-trained transformer model for WSI classification. Such pre-training has been shown to transfer to other modalities, and we corroborate this finding in three digital pathology tasks. To do so, we use a standalone layer for sequence reduction aimed to overcome common challenges in WSI classification with transformer architectures, and to reduce the compute budget required for processing large inputs with deep self-attention-based architectures.

Our SeqShort layer was developed with the hypothesis that there is redundant visual information in the full sequence of patches of a WSI, similar to the redundancy in text sequences previously explored in the NLP literature^26–32^. Our results in section 2.4 show that high-attention patches are preserved throughout the different learned queries, indicating their importance for the prediction. Indeed 99.7% of the pairs of patch rankings based on different query vectors show correlation coefficient $> 0.7$. These results suggest that there is redundancy in the full sequence of patches, as certain patches are consistently more important than others, and that classification is possible by aggregating them into a shorter sequence. Moreover, in section 2.3, we show that classification performance is increased between 0.017 and 0.038 when the downstream classifier is augmented with positional embeddings that encodes the sequential order in the output generated by the SeqShort layer. For the sake of simplicity, most of our experiments are done considering a WSI to be an unordered bag of image patches. However, including patch location positional encoding is compatible with our approach, producing a further performance boost.

Text pre-trained transformers have been proven successful in non-language related tasks^33,35–37^. In section 2.2 we show that classification performance can be increased by up to 0.079 AUROC points just by fine-tuning less than 0.1% of the parameters of a deep transformer that was pre-trained on a large text dataset, compared to the same model trained from scratch. The best performing model in our experiments is RoBERTa-base, which outperformed BERT-base in the WSI classification, reflecting these models’ performance difference in several NLP tasks. Notably, these models have the same architecture but differ in the pre-training objective and dataset size. Only the ALBERT-base LM was outperformed by the model trained from scratch. In contrast to the rest of the models in this experiment, ALBERT-base contains a single fully trainable layer whose parameters are reused in the subsequent layers, which might explain its lower capacity of transferring to other domains. These results suggest that both the transformer size and text corpus volume play a role in the model performance in a WSI classification task.

Our primary goal is not to design a novel MIL algorithm that surpasses state-of-the-art, but rather to demonstrate that out-of-the-box LMs can transfer their representations of sequential data to the field of digital pathology. Models designed for this discipline are very performant, have a parameter count orders of magnitude smaller than LMs, and inference time considerably faster. We consider it a reasonable decision to employ WSI MIL classifiers instead of models that were designed and trained for NLP. In section 2.1 our LM-based approach outperforms the WSI-dedicated methods in most LNM and IBC experiments, and showing competitive AUROC in RCC classification. The experiments using the CTransPath feature extractor show a general increase of performance for all models, and is only detrimental for our model when using 25% of the data in the LNM task. These results show that LMs are competitive WSI classifiers, outperforming MIL models in some of the tasks, and suggesting that this direction of research in digital pathology is worth exploring further.

The scope of this work is limited to “base” LMs that comprise 80 million parameters, and are possible to fit in a single GPU. We included an experiment with Llama3-8B^49^ in table 2. With 8 billion parameters, we could only fine-tune it using 8-bit quantized model weights, making this experiment not directly comparable to the rest of the models in the comparison. A natural extension of our study is to do further experiments with Large Language Models such as OPT-175B, with 175 billion parameters^50^, or the rest of the Llama family of models that comprise up to 405 billion parameters^49^.

## Methods

### Sequence shortening

Existing methods^11,27,51^ for sequence reduction are not suitable for MIL WSI problems. Since there is no spatial information about instances in an unordered bag, concatenating neighboring feature vectors or taking their strided average is meaningless, as the order of the patches in a bag is arbitrary. Methods that employ a linear projection for dimensionality reduction after instance concatenation or sequence reshaping are not applicable to WSIs either, as they require a fixed and known input shape.

Here we propose using MHA for sequence shortening. Similar ideas have been explored in text-vision multi-modal understanding tasks^52–54^, and is reminiscent of how object queries are used in transformer object detection^55^, with the advantage of not requiring object-level annotations.

Given $\varvec{X} \in \mathbb {R}^{M\times d}$ the sequence of $M$ $d$-dimensional feature vectors of non-overlapping WSI tiles, we introduce our *SeqShort* input layer that generates a new sequence $\varvec{X_{S}} \in \mathbb {R}^{S\times h}$ with an MHA layer:

2 $$\begin{aligned} \begin{aligned} \varvec{X_{S}}&= \text{ MHA }\left(Q=\varvec{Q_{l}},K=\varvec{X},V=\varvec{X}\right) + \varvec{Q_{l}} \\&= \text{ Concat }(head_1,..., head_k) \varvec{W^O} + \varvec{Q_{l}}, \end{aligned} \end{aligned}$$

with

3 $$\begin{aligned} \begin{aligned} head_i = \text{ Attention }\left(Q=\varvec{Q_{l}}\varvec{W_i^Q},K=\varvec{X}\varvec{W_i^K}, V=\varvec{X}\varvec{W_i^V}\right), \\ \text{ Attention }(Q, K, V) = \text{ softmax }(QK^T / \sqrt{d_h})V, \end{aligned} \end{aligned}$$

where $\varvec{Q_{l}} \in \mathbb {R}^{S\times h}$ is a learnable sequence of $S$ $h$-dimensional query vectors, the matrices $\varvec{W}$ are learnable linear projections, $d_h$ is a scaling factor commonly set as the layer’s hidden dimension, and *k* is the number of attention heads of the layer. Both $S$ and $h$ are hyperparameters independent of the shape of the original sequence $\varvec{X}$, and it is $S$ which defines the output sequence length of the MHA operation in SeqShort.

This MHA operation has a sorting effect: independent of the arrangement of the patch feature vectors in $\varvec{X}$, the first row of $\varvec{X_{S}}$ aggregates the instances that the first query vector in $\varvec{Q_{l}}$ agrees with the most; the second row of $\varvec{X_{S}}$ aggregates the instances that the second query vector in $\varvec{Q_{l}}$ agrees with the most, and so on. This enables to incorporate positional information in our model based on a new interpretation: instead of thinking of the original arrangement of instances in the WSI 2D space, we consider the order of the rows of $\varvec{X_{S}}$ as the available positional information possible to encode.

The resulting time complexity of the MHA operation performed by our input layer is $O(n)$ because of the fixed-size $\varvec{Q_{l}}$, and since SeqShort is a single layer, the main compute load lies in the subsequent deeper transformer model in our pipeline. Although our method does not change the computational complexity of the MHSA layers of the transformer itself, by performing sequence reduction, the amount of FLOPs and memory it requires becomes constant with respect to the original number of WSI patches. The result is an overall considerable reduction of computational cost. Fig. 1c visualizes how the required FLOPs per forward pass scale better when using the SeqShort layer. For example, the average WSI in the IBC dataset comprises 7690 patches, which takes 734ms to be processed with a BERT-base encoder using our hardware. This time is reduced to 14ms when SeqShort is used as input layer.

### Transformer models

In our experiments we find that a BERT-base encoder^6^ pre-trained with the masked language modeling task of *Robustly optimized BERT pre-training Approach* (RoBERTa)^8^ on a corpora of more than 160GB of uncompressed text comprised by BookCorpus^56^, CC-News^57^, OpenWebText^58^ and Stories^59^ yields the best results. We discard the vocabulary embeddings lookup table of RoBERTa-base as it is not needed for weakly supervised image classification.

### Complete pipeline

Our pipeline is illustrated in Fig. 1. As a pre-processing step, we extract non-overlapping tissue tiles of $256 \times 256$ pixels from each WSI. Tissue segmentation is done as in^60^. We use $\times$20 and $\times$10 magnification in different experiments. We generate the instance-level feature vectors using an EfficientNetV2-L^43^ pre-trained on ImageNet^45^.

The complete weakly supervised architecture that performs classification on the bag of instance vectors is composed of the SeqShort layer and a transformer language model. We set the vector embedding dimension of SeqShort to $h=768$ (the hidden dimension of the used transformers), and $k=4$ attention heads. For the lymph node classification task we set the output length of SeqShort to $S=511$, and for the cancer subtyping tasks, to $S=256$. A learnable [CLS] token is concatenated to the output of SeqShort, and added a sequence of learnable positional embeddings. The last hidden representation of [CLS] is the input of a multilayer perceptron (MLP) classification head. Altogether, our model comprises a total of 3.3M trainable parameters.

### Implementation and training

The method was implemented in Python, using PyTorch^61^ as deep learning back-end. The pre-trained weights of EfficientNetV2-L and RoBERTa were downloaded from Torchvision^62^ and HuggingFace^63^, respectively. Training of our models was done with the aid of PyTorch-Lightning^64^, on a single NVIDIA Tesla V100 GPU. The code of this project is available at https://github.com/bozeklab/lmagp/.

All our models were trained for 200 epochs. For the lymph node classification task the first 5 epochs were used as learning rate warm-up stage, followed by one cycle of a cosine schedule, with a maximum learning rate of $1\times 10^{-4}$, and batch size of 16. For the cancer subtyping tasks, the warm-up stage lasted 10 epochs, followed by two cycles of a cosine schedule, with a maximum learning rate of $5\times 10^{-5}$, and batch size of 32. Adam^65^ was used as optimization algorithm.

### Datasets

#### Lymph node metastases classification

For this task we used the dataset provided by the CAMELYON16 grand challenge (https://camelyon16.grand-challenge.org/) which comprises 400 Hematoxylin and Eosin (H&E) stained WSIs of sentinel lymph nodes of breast cancer patients, scanned by 3DHISTECH and Hamamatsu scanners at $\times 40$ at the Radboud University Medical Center and the University Medical Center Utrecht, Netherlands. The grand challenge dataset is divided in a train set of 270 WSIs (160 normal slides, and 110 slides containing metastases), and a test set of 129 WSIs (80 normal slides, 49 slides containing metastases). In our experiments, we divided the provided train set in 90%/10% stratified splits for training and validation, respectively.

#### Invasive breast carcinoma subtype classification

We use a subset of 1,038 H&E stained WSIs from the TCGA-BRCA project within The Cancer Genome Atlas repository (https://www.cancer.gov/tcga). Out of the 1,038 slides, 889 were of patients with Invasive Ductal Carcinoma, and 149 were of patients with Invasive Lobular Carcinoma. We follow the study design in^40,44^ and evaluate the models using stratified 10-fold cross-validation on patient level.

#### Renal cell carcinoma subtype classification

We use 918 H&E stained WSIs of Renal Cell Carcinoma cases from the TCGA repository. Out of these samples, 289 were of Chromophobe Cell Carcinoma patients, 118 were of Papillary Cell Carcinoma patients, and 498 were of Clear Cell Carcinoma patients, coming from the TCGA-KICH, TCGA-KIRP and TCGA-KIRC projects, respectively. We follow the same study design as in the IBC subtype classification task, and evaluate the models using stratified 10-fold cross-validation on patient level.

## Supplementary Information

Supplementary Information.

## Data Availability

Data used in this article comes from The Cancer Genome Atlas (https://portal.gdc.cancer.gov/) and the CAMELYON16 Grand Challenge (https://camelyon16.grand-challenge.org/).

## References

[1] FAIR (Last accessed: 12.09.2024) fvcore library. https://github.com/facebookresearch/fvcore/.

[2] Vaswani, A. et al. Attention is all you need. *NeurIPS* **2017**, 30 (2017).

[3] Ott, M. et al. Scaling neural machine translation. In *WMT 2018* 1–9 (Association for Computational Linguistics, 2018).

[4] So, D., Le, Q., Liang, C. The evolved transformer. In: ICML 2019, PMLR, pp 5877–5886 (2019).

[5] Dehghani, M., Gouws, S., Vinyals, O., et al. Universal transformers. 10.48550/ARXIV.1807.03819 (2018).

[6] Devlin, J. et al. BERT: Pre-training of deep bidirectional transformers for language understanding. In *NAACL-HLT* 4171–4186 (Association for Computational Linguistics, Minneapolis, Minnesota, 2019).

[7] Lan, Z., Chen, M., Goodman, S., et al. ALBERT: A lite BERT for self-supervised learning of language representations. In: ICLR (2020).

[8] Liu, Y., Ott, M., Goyal, N., et al. Roberta: A robustly optimized BERT pretraining approach. CoRR abs/1907.11692 (2019).

[9] Radford, A., Narasimhan, K., Salimans, T., et al. Improving language understanding by generative pre-training. (2018).

[10] Dosovitskiy, A., Beyer, L., Kolesnikov, A., et al. An image is worth 16x16 words: Transformers for image recognition at scale. In: ICLR (2021).

[11] Liu, Z., Lin, Y., Cao, Y., et al. Swin transformer: Hierarchical vision transformer using shifted windows. In: ICCV. IEEE, pp 9992–10002 (2021).

[12] Zhai, X., Kolesnikov, A., Houlsby, N., et al. Scaling vision transformers. In: CVPR. IEEE, pp 1204–1213 (2022).

[13] Zheng, S. et al. Rethinking semantic segmentation from a sequence-to-sequence perspective with transformers. In *CVPR. Computer Vision Foundation* (ed. Zheng, S.) 6881–6890 (IEEE, 2021).

[14] Ramesh, A., Pavlov, M., Goh, G., et al. Zero-shot text-to-image generation. In: Meila M, Zhang T (eds) ICML, PMLR, vol 139. PMLR, pp 8821–8831 (2021).

[15] Saharia, C., Chan, W., Saxena, S., et al. Photorealistic text-to-image diffusion models with deep language understanding. CoRR abs/2205.11487 (2022).

[16] Dietterich, T. G., Lathrop, R. H. & Lozano-Pérez, T. Solving the multiple instance problem with axis-parallel rectangles. *Artif. Intell.* **89**(1–2), 31–71 (1997).

[17] Maron, O. & Lozano-Pérez, T. A framework for multiple-instance learning. In *NeurIPS* (eds Jordan, M. I. et al.) 570–576 (The MIT Press, 1997).

[18] Reisenbüchler, D. et al. Local attention graph-based transformer for multi-target genetic alteration prediction. In *MICCAI 2022, Part II, LNCS* Vol. 13432 (eds Wang, L., Dou, Q., Fletcher, P. T. et al.) 377–386 (Springer, 2022).

[19] Lv, Z. et al. Joint region-attention and multi-scale transformer for microsatellite instability detection from whole slide images in gastrointestinal cancer. In *MICCAI 2022, Part II* Vol. 13432 (eds Wang, L., Dou, Q., Fletcher, P. T. et al.) 293–302 (Springer, 2022).

[20] Shao, Z., Bian, H., Chen, Y., et al. Transmil: Transformer based correlated multiple instance learning for whole slide image classification. In: Ranzato M, Beygelzimer A, Dauphin YN, et al (eds) NeurIPS 2021, pp 2136–2147 (2021).

[21] Li, H., Yang, F., Zhao, Y., et al. DT-MIL: deformable transformer for multi-instance learning on histopathological image. In: de Bruijne M, Cattin PC, Cotin S, et al (eds) MICCAI 2021, Part VIII, LNCS, vol 12908. Springer, pp 206–216 (2021).

[22] Zhao, Y. et al. SETMIL: spatial encoding transformer-based multiple instance learning for pathological image analysis. In *Medical Image Computing and Computer Assisted Intervention - MICCAI 2022–25th International Conference, Singapore, September 18–22, 2022, Proceedings, Part II, LNCS* Vol. 13432 (eds Wang, L., Dou, Q., Fletcher, P. T. et al.) 66–76 (Springer, 2022).

[23] Shao, Z. et al. LNPL-MIL: learning from noisy pseudo labels for promoting multiple instance learning in whole slide image. In *IEEE/CVF International Conference on Computer Vision, ICCV 2023, Paris, France, October 1–6, 2023* (ed. Shao, Z.) 21438 (IEEE, 2023).

[24] Tang, W. et al. Feature re-embedding: Towards foundation model-level performance in computational pathology. In *IEEE/CVF Conference on Computer Vision and Pattern Recognition, CVPR 2024, Seattle, WA, USA, June 16–22, 2024* (ed. Tang, W.) 11343–11352 (IEEE, 2024).

[25] Zheng, Y. et al. A graph-transformer for whole slide image classification. *IEEE Trans. Med. Imaging* **41**(11), 3003–3015 (2022). 35594209 10.1109/TMI.2022.3176598 PMC9670036

[26] Wang, S., Li, BZ., Khabsa, M., et al. Linformer: Self-attention with linear complexity. CoRR abs/2006.04768 (2020).

[27] Dai, Z., Lai, G., Yang, Y., et al. Funnel-transformer: Filtering out sequential redundancy for efficient language processing. In: Larochelle H, Ranzato M, Hadsell R, et al (eds) NeurIPS 2020 (2020).

[28] Clark, K., Khandelwal, U., Levy, O., et al. What does BERT look at? an analysis of bert’s attention. In: Linzen T, Chrupala G, Belinkov Y, et al (eds) ACL 2019. Association for Computational Linguistics, pp 276–286 (2019).

[29] Beltagy, I., Peters, ME., Cohan, A. Longformer: The long-document transformer. CoRR abs/2004.05150 (2020).

[30] Zaheer, M., Guruganesh, G., Dubey, KA., et al. Big bird: Transformers for longer sequences. In: Larochelle H, Ranzato M, Hadsell R, et al (eds) NeurIPS 2020 (2020).

[31] Roy, A. et al. Efficient content-based sparse attention with routing transformers. *Trans. Assoc. Comput. Linguistics* **9**, 53–68 (2021).

[32] Qiu, J. et al. Blockwise self-attention for long document understanding. In *EMNLP 2020, Findings of ACL, vol EMNLP 2020* (eds Cohn, T. et al.) 2555–2565 (Association for Computational Linguistics, 2020).

[33] Cobbe, K., Kosaraju, V., Bavarian, M., et al. Training verifiers to solve math word problems. CoRR abs/2110.14168 (2021).

[34] Deletang, G., Ruoss, A., Duquenne, PA., et al. Language modeling is compression. In: The Twelfth International Conference on Learning Representations (2024).

[35] Huang, W., Abbeel, P., Pathak, D., et al. Language models as zero-shot planners: Extracting actionable knowledge for embodied agents. In: Chaudhuri K, Jegelka S, Song L, et al (eds) ICML 2022, PMLR, vol 162. PMLR, pp 9118–9147 (2022).

[36] Ilharco, G., Zellers, R., Farhadi, A., et al. Probing contextual language models for common ground with visual representations. In: Toutanova K, Rumshisky A, Zettlemoyer L, et al (eds) NAACL-HLT 2021. Association for Computational Linguistics, pp 5367–5377 (2021).

[37] Lu, K., Grover, A., Abbeel, P., et al. Frozen pretrained transformers as universal computation engines. In: AAAI-IAAI-EAAI 2022. AAAI Press, pp 7628–7636 (2022).

[38] Abnar, S., Zuidema, W.H. Quantifying attention flow in transformers. In: Jurafsky D, Chai J, Schluter N, et al (eds) ACL 2020. Association for Computational Linguistics, pp 4190–4197 (2020).

[39] Ilse, M., Tomczak, JM., Welling, M. (2018) Attention-based deep multiple instance learning. In: Dy JG, Krause A (eds) ICML 2018, PMLR, vol 80. PMLR, pp 2132–2141

[40] Lu, M. Y. et al. Data-efficient and weakly supervised computational pathology on whole-slide images. *Nat. Biomed. Eng.* **5**(6), 555–570 (2021). 33649564 10.1038/s41551-020-00682-w PMC8711640

[41] Li, B., Li, Y., Eliceiri, KW. Dual-stream multiple instance learning network for whole slide image classification with self-supervised contrastive learning. In: CVPR 2021. Computer Vision Foundation / IEEE, pp 14318–14328 (2021). 10.1109/CVPR46437.2021.01409 PMC8765709 35047230

[42] Wagner, S. J. et al. Transformer-based biomarker prediction from colorectal cancer histology: A large-scale multicentric study. *Cancer Cell* **41**(9), 1650-1661.e4 (2023). 37652006 10.1016/j.ccell.2023.08.002 PMC10507381

[43] Tan, M. & Le, Q. V. Efficientnetv2: Smaller models and faster training. In *ICML 2021* Vol. 139 (eds Meila, M. & Zhang, T.) 10096–10106 (PMLR, 2021).

[44] Chen, RJ., Chen, C., Li, Y., et al. Scaling vision transformers to gigapixel images via hierarchical self-supervised learning. In: CVPR 2022. IEEE, pp 16123–16134 (2022).

[45] Deng, J., Dong, W., Socher, R., et al. Imagenet: A large-scale hierarchical image database. In: CVPR 2009, Ieee, pp 248–255 (2009).

[46] Wang, X. et al. Transformer-based unsupervised contrastive learning for histopathological image classification. *Med. Image Anal.* **81**, 102559. 10.1016/j.media.2022.102559 (2022). 35952419 10.1016/j.media.2022.102559

[47] Yang, Z. et al. Xlnet: Generalized autoregressive pretraining for language understanding. *NeurIPS* **2019**, 32 (2019).

[48] Radford, A. et al. Language models are unsupervised multitask learners. *OpenAI blog* **1**(8), 9 (2019).

[49] Dubey, A., Jauhri, A., Pandey, A., et al. The llama 3 herd of models. arXiv preprint arXiv:2407.21783 (2024).

[50] Zhang, S., Roller, S., Goyal, N., et al. OPT: open pre-trained transformer language models. CoRR abs/2205.01068. 10.48550/ARXIV.2205.01068 (2022).

[51] Nawrot, P. et al. Hierarchical transformers are more efficient language models. In *NAACL 2022* (eds Carpuat, M. et al.) 1559–1571 (Association for Computational Linguistics, 2022).

[52] Liu, Y., Li, L., Zhang, B., et al. Matcr: Modality-aligned thought chain reasoning for multimodal task-oriented dialogue generation. In: El-Saddik A, Mei T, Cucchiara R, et al (eds) Proceedings of the 31st ACM International Conference on Multimedia, MM 2023, Ottawa, ON, Canada, 29 October 2023- 3 November 2023. ACM, pp 5776–5785 (2023).

[53] Zhang, H., Li, X. & Bing, L. Video-llama: An instruction-tuned audio-visual language model for video understanding. In *Proceedings of the 2023 Conference on Empirical Methods in Natural Language Processing, EMNLP 2023 - System Demonstrations, Singapore, December 6–10, 2023* (eds Feng, Y. & Lefever, E.) 543–553 (Association for Computational Linguistics, 2023).

[54] Li, J., Li, D., Savarese, S., et al. BLIP-2: bootstrapping language-image pre-training with frozen image encoders and large language models. In: Krause A, Brunskill E, Cho K, et al (eds) International Conference on Machine Learning, ICML 2023, 23-29 July 2023, Honolulu, Hawaii, USA, Proceedings of Machine Learning Research, vol 202. PMLR, pp 19730–19742 (2023).

[55] Carion, N. et al. End-to-end object detection with transformers. In *Computer Vision - ECCV 2020–16th European Conference, Glasgow, UK, August 23–28, 2020, Proceedings, Part I* Vol. 12346 (eds Vedaldi, A., Bischof, H., Brox, T. et al.) 213–229 (Springer, 2020).

[56] Zhu, Y., Kiros, R., Zemel, RS., et al. Aligning books and movies: Towards story-like visual explanations by watching movies and reading books. In: ICCV 2015. IEEE Computer Society, pp 19–27 (2015).

[57] Mackenzie, JM., Benham, R., Petri, M., et al. Cc-news-en: A large english news corpus. In: d’Aquin M, Dietze S, Hauff C, et al (eds) CIKM 2020. ACM, pp 3077–3084 (2020).

[58] Gokaslan, A., Cohen, V. Openwebtext corpus. http://Skylion007.github.io/OpenWebTextCorpus (2019).

[59] Trinh, TH., Le, QV. A simple method for commonsense reasoning. CoRR abs/1806.02847 (2018).

[60] Graham, S. et al. Hover-net: Simultaneous segmentation and classification of nuclei in multi-tissue histology images. *Med. Image Anal.* **58**, 101563 (2019). 31561183 10.1016/j.media.2019.101563

[61] Paszke, A., Gross, S., Massa, F., et al. Pytorch: An imperative style, high-performance deep learning library. In: Wallach HM, Larochelle H, Beygelzimer A, et al (eds) Advances in Neural Information Processing Systems 32: Annual Conference on Neural Information Processing Systems 2019, NeurIPS 2019, December 8-14, 2019, Vancouver, BC, Canada, pp 8024–8035, https://proceedings.neurips.cc/paper/2019/hash/bdbca288fee7f92f2bfa9f7012727740-Abstract.html (2019).

[62] Marcel, S., Rodriguez, Y. Torchvision the machine-vision package of torch. In: Bimbo AD, Chang S, Smeulders AWM (eds) Proceedings of the 18th International Conference on Multimedia 2010, Firenze, Italy, October 25-29, 2010. ACM, pp 1485–1488. 10.1145/1873951.1874254 (2010).

[63] Wolf, T., Debut, L., Sanh, V., et al. Huggingface’s transformers: State-of-the-art natural language processing. CoRR abs/1910.03771. http://arxiv.org/abs/1910.03771,1910.03771 (2019).

[64] Falcon, W. The PyTorch Lightning team. PyTorch Lightning.10.5281/zenodo.3828935, https://github.com/Lightning-AI/lightning (2019).

[65] Kingma, DP., Ba, J. Adam: A method for stochastic optimization. In: Bengio Y, LeCun Y (eds) 3rd International Conference on Learning Representations, ICLR 2015, San Diego, CA, USA, May 7-9, 2015, Conference Track Proceedings, http://arxiv.org/abs/1412.6980 (2015).

