# Supplementary material for: Efficient WSI classification with sequence reduction and transformers pretrained on text
Source: Sci Rep. 2025 Feb 15;15:5612. doi: 10.1038/s41598-025-88139-5 (PMC11829941; doi:10.1038/s41598-025-88139-5)
Supplement: Supplementary file 1 — Supplementary Information. [file 41598_2025_88139_MOESM1_ESM.pdf]

Supplementary material

Supplementary Table 1: Performance of different MIL algorithms in LNM and IBC classification tasks using CTransPath features [6]. Best and second best classification results are in **bold** and <u>underlined</u>, respectively.

Method	x10 magnification		x20 magnification	
	25% train set	100% train set	25% train set	100% train set
**Lymph Node Metastases classification**				
ABMIL [1]	**0.881**	<u>0.910</u>	**0.938**	<u>0.971</u>
CLAM [3]	<u>0.695</u>	**0.928**	0.717	0.953
DS-MIL [2]	0.541	0.744	0.521	0.934
TransMIL [4]	0.634	0.870	0.654	0.935
Wagner et al. [5]	0.663	**0.928**	<u>0.757</u>	**0.974**
**Ours**	0.512	0.783	0.448	0.957
**Invasive Breast Carcinoma subtype classification**				
ABMIL [1]	0.868 ± 0.062	0.896 ± 0.066	0.690 ± 0.298	0.893 ± 0.057
CLAM [3]	**0.921 ± 0.065**	0.929 ± 0.033	<u>0.895 ± 0.051</u>	**0.937 ± 0.300**
DS-MIL [2]	<u>0.913 ± 0.056</u>	<u>0.934 ± 0.037</u>	**0.903 ± 0.050**	<u>0.934 ± 0.036</u>
TransMIL [4]	0.890 ± 0.060	<u>0.934 ± 0.043</u>	0.882 ± 0.061	0.924 ± 0.042
Wagner et al. [5]	0.903 ± 0.059	**0.935 ± 0.035**	0.881 ± 0.061	0.927 ± 0.052
**Ours**	0.860 ± 0.087	0.928 ± 0.043	0.860± 0.062	0.914 ± 0.057

References

[1] Ilse M, Tomczak JM, Welling M (2018) Attention-based deep multiple instance learning. In: Dy JG, Krause A (eds) ICML 2018, PMLR, vol 80. PMLR, pp 2132–2141

[2] Li B, Li Y, Eliceiri KW (2021) Dual-stream multiple instance learning network for whole slide image classification with self-supervised contrastive learning. In: CVPR 2021. Computer Vision Foundation / IEEE, pp 14318–14328

[3] Lu MY, Williamson DF, Chen TY, et al (2021) Data-efficient and weakly supervised computational pathology on whole-slide images. Nature biomedical engineering 5(6):555–570

[4] Shao Z, Bian H, Chen Y, et al (2021) Transmil: Transformer based correlated multiple instance learning for whole slide image classification. In: Ranzato M, Beygelzimer A, Dauphin YN, et al (eds) NeurIPS 2021, pp 2136–2147

[5] Wagner SJ, Reisenbüchler D, West NP, et al (2023) Transformer-based biomarker prediction from colorectal cancer histology: A large-scale multicentric study. Cancer Cell 41(9):1650–1661.e4. https://doi.org/https://doi.org/10.1016/j.ccell.2023.08.002, URL https://www.sciencedirect.com/science/article/pii/S1535610823002787

[6] Wang X, Yang S, Zhang J, et al (2022) Transformer-based unsupervised contrastive learning for histopathological image classification. Medical Image Analysis 81:102559. https://doi.org/https://doi.org/10.1016/j.media.2022.102559, URL https://www.sciencedirect.com/science/article/pii/S1361841522002043
